# Paroxysmal eruptions tracked by variations of helium isotopes: inferences from Piton de la Fournaise (La Réunion island)

**DOI:** 10.1038/s41598-020-66260-x

**Published:** 2020-06-17

**Authors:** G. Boudoire, A. L. Rizzo, I. Arienzo, A. Di Muro

**Affiliations:** 1 Université Clermont Auvergne, CNRS, IRD, OPGC, Laboratoire Magmas et Volcans, 6 avenue Blaise Pascal, 63178 Aubière, France; 2 Istituto Nazionale di Geofisica e Vulcanologia, Sezione di Palermo, Via Ugo La Malfa 153, 90146 Palermo, Italy; 3 Dipartimento di Fisica e Scienze della Terra, Università degli Studi di Ferrara, 44121 Ferrara, Italy; 4 Istituto Nazionale Di Geofisica e Vulcanologia, Osservatorio Vesuviano, Via Diocleziano 328, 80124 Napoli, Italy; 5 Université de Paris, Institut de Physique du Globe de Paris, CNRS, F-75005 Paris, France; 6 Observatoire Volcanologique du Piton de la Fournaise, Institut de Physique du Globe de Paris, F-97418 La Plaine des Cafres, France

**Keywords:** Natural hazards, Geochemistry, Volcanology

## Abstract

Helium (He) with its isotopes (^3^He, ^4^He) is a key tracer enabling the Earth’s mantle and dynamics to be characterized. Enrichment in primordial helium (^3^He) has been detected in volcanic gases of numerous magmatic systems in different geodynamic settings. Despite past use to monitor volcano-tectonic unrest, temporal ^3^He/^4^He variability in volcanic emissions is still poorly constrained. Here, we investigate noble gas chemistry of Piton de la Fournaise hotspot volcano, where temporal fluctuations of ^3^He/^4^He in response to the eruptive activity have never been studied. We compare the ^3^He/^4^He signature of volcanic gases and fluid inclusions and we highlight analogous evolution of the ^3^He/^4^He signature in both during the last decades of eruptive activity (1990–2017), even during the same eruption. We show that the maximum enrichment in ^3^He is found in magmatic fluids that fed the most voluminous eruptions which culminated in caldera collapse events. We argue that this enrichment in ^3^He mostly reflects a greater contribution of magmatic fluids from a primitive component of the mantle plume. These results emphasize that He isotopes may provide warnings of increases in deep magmatic contributions that potentially herald paroxysmal eruptions, as documented here at Piton de la Fournaise (2007) and also at Kilauea (2018).

## Introduction

Noble gases are known to provide important clues on Earth’s genesis, reservoirs and current dynamics^1–4^. In particular, the isotopes of helium (He) are of particular interest due to the primordial origin of ^3^He with respect to ^4^He that is continuously produced by the radioactive decay of U-Th that have distinct proportions in Earth’s reservoirs^5,6^. Coupled with other noble gases characterised by distinct physical-chemical properties, He is also useful to characterize mantle metasomatism, recycling of crustal material in the mantle, diffusive fractionation, melt evolution during ascent and aging-degassing^7–11^. In volcanic contexts, long- to short-term temporal variability of He isotope signature has been documented in (i) gas trapped in fluid inclusions (FI) in mafic minerals and in (ii) volcanic gases. In the former case variations in ^3^He/^4^He were attributed to the variable influence of crustal and mantle components in magmatic melts^12–18^, whilst in the latter case it was considered to be related to time-changing contribution of magmatic fluids with respect to crustal sources or to the arrival of deep undegassed magma^19–25^. In this respect, recent studies have stressed the relevance of ^3^He/^4^He monitoring in volcanic gases to detect a range of unrest conditions from magmatic to phreato-magmatic^16,18,19,21–23,25,26^. With the exception of a few intra-plate volcanoes on Earth (e.g., Etna, El Hierro), most of the studies of ^3^He/^4^He monitoring volcanic gases have been carried out in subduction-related settings (e.g., Stromboli, Santorini, Turrialba, Ontake) where long quiescent periods and/or the emission of variably differentiated lavas, often free of gas-rich fluid inclusions in mafic minerals, are common^18,20–23^. This makes comparing ^3^He/^4^He in volcanic gases and FI challenging at active volcanoes, even if it could potentially shed light on the ongoing magmatic dynamics as well as on magma residence and crystallization timing and allow the forecasting of unrest phases. Not surprisingly, detailed and innovative studies based on this approach have been totally missing until present.

**Figure 1:**
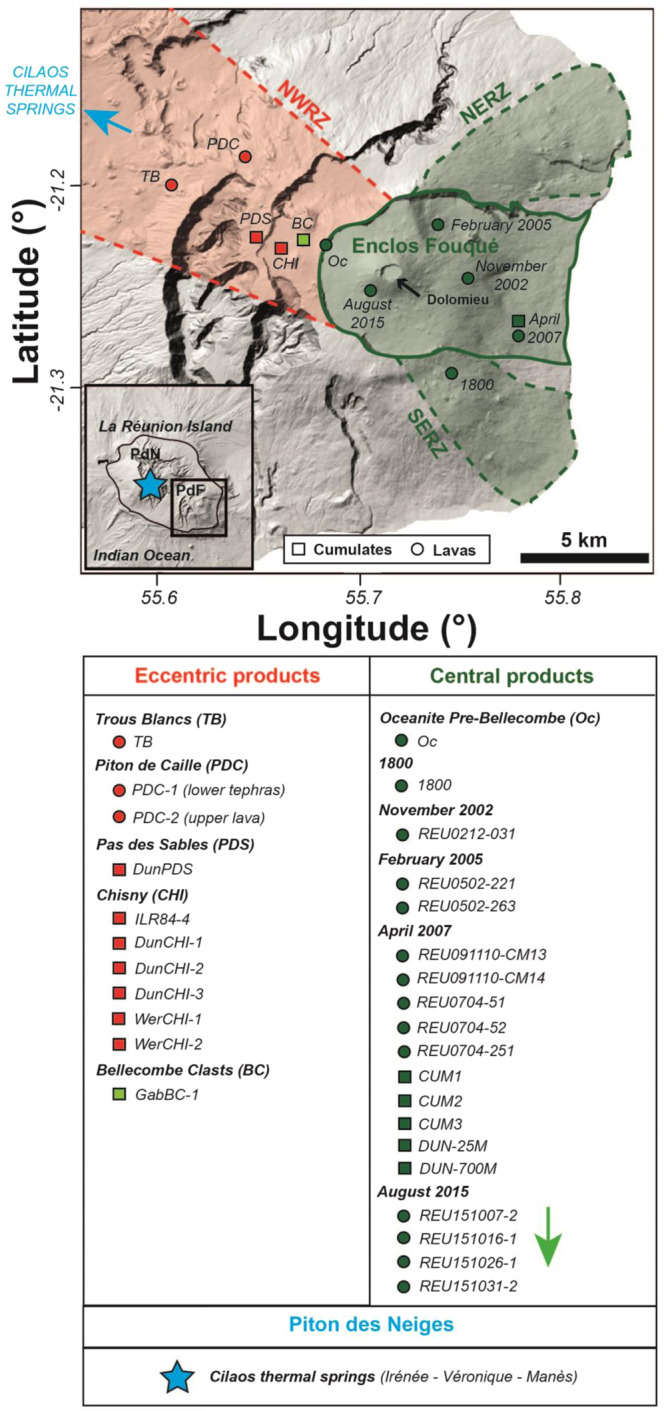
Location map of sampled sites at La Réunion (PdF for Piton de la Fournaise and PdN for Piton des Neiges volcanoes). Crushed crystals from lavas (circles) and cumulates (squares) in the NW rift zone (NWRZ; in red) and in the Enclos Fouqué caldera (EF; in green) and related NE-SE rift zones (NERZ, SERZ; in green). The green arrow represents the chronology of the products from the August 2015 eruption described in this study. Volcanic gases sampled in Cilaos thermal springs at PdN (in blue). Analytical protocols are reported in the Methods.

Here, we aim to compare He systematics in FI in crystals from lavas and cumulates (Table S1) of Piton de la Fournaise (PdF) with (i) Sr isotopes of host rocks/crystals (Table S1) and (ii) He isotopes in volcanic gases from thermal springs (Table S2) from Piton des Neiges (PdN). Piton des Neiges represents the largest (and oldest) volcanic edifice of La Réunion island, with the only permanent CO_2_-rich thermal springs^10,27^. The Piton de la Fournaise edifice has progressively built up on PdN flank since about 0.5 Ma^28^. Since the beginning of the 18^th^ century, activity at PdF has been characterized by frequent and, on average, short-lived (hours-to-weeks, rarely months) eruptions^29,30^. The high frequency of PdF eruptions allows the investigation of fast ^3^He/^4^He fluctuations in response to the eruptive activity over short time scales (e.g., days to years). La Réunion island is also set in a hotspot-related context^31^ favouring the emission of basalts containing an abundance of mafic minerals that are enriched in gas entrapped in FI with respect to lavas emitted in subduction-related contexts^10,21^. This last point is crucial to investigate the composition of gases in FI and to compare it with volcanic gases. Geochemically, a distinction is classically made between eccentric and central lavas emitted at PdF^32,33^. The former are sporadically emitted along the North West rift zone connecting PdN and PdF volcanoes (NWRZ; Fig. 1) and ascend rapidly from the mantle and the mantle-crust underplating layer^33^. The latter are emitted at a high frequency (with an average of one eruption every 9 months) inside the Enclos Fouqué caldera and along the South-East and North-East rift zones (SERZ and NERZ; Fig. 1) and are classically related to the shallower, crustal plumbing system of PdF^10,32–34^. Note that the Enclos Fouqué caldera (8 × 13 km) results from the most violent explosive activity (“Bellecombe” events) of PdF of the last 5 kyr^35^. The endmembers of the three datasets used in this study (He isotopes in FI, Sr isotopes from host rocks/crystals and He isotopes in volcanic gases) are detailed in Table 1 (see Table S1 for full data set).

**Table 1:** Characteristics (sample type, gas contents and ratios, He-Sr isotopes) of the main end-members selected from the Table S1 and discussed in this study. Ages and inferred crystallization depths^10^. In bold, minimum and maximum values for each parameter. ‘TGC’ for Total Gas Content.

Samples information						Gas contents and ratios					Isotopes ratios	
Location	Type	Material	Eruption/Site	Emission date	Name	TGC (mol/g)	^4^He (mol/g)	^4^He/^40^Ar*	He/CO_2_	Inferred crystallisation depth	Rc/Ra	^87^Sr/^86^Sr
Central	Lavas	Crystals	February 2005	February 2, 2005	REU0502-221	**9.6E-10**	**9.8E-13**	**4.9**	**1.0E-03**	**Oceanic crust**	**12.5**	0.704181
Central	Lavas	Crystals	August 2015	October 26, 2015	REU151026-1	1.1E-09	3.2E-13	3.5	3.9E-04		**14.3**	0.704165
Peripheral	Lavas	Crystals	Piton de Caille	<5 kyrs	PDC-1	1.0E-07	7.1E-12	**1.3**	7.0E-05	Upper mantle: underplating layer	13.8	**0.704104**
Peripheral	Lavas	Crystals	Trous Blancs	<10 krys	TB	2.9E-08	4.9E-12	2.0	1.7E-04		13.4	**0.704261**
Peripheral	Cumulates	Crystals	Chisny	≈ 381 BP	ILR84-4	**7.8E-07**	**2.9E-11**	1.4	3.7E-05	**Upper mantle: deep melt horizon**	12.8	
Peripheral	Spring waters	Free gas	Cilaos (Irénée)	April 12, 2017	IRE170412-1			1.4	**1.2E-05**	Upper mantle	13.6	
Peripheral	Spring waters	Free gas	Cilaos (Véronique)	April 12, 2017	VER170412-1			1.8	5.7E-5		14.0	
Peripheral	Spring waters	Free gas	Cilaos (Manès)	April 12, 2017	MAN170412-2			2.2	3.3E-5		13.9	

### Helium isotope variability in the plumbing system

**Figure 2:**
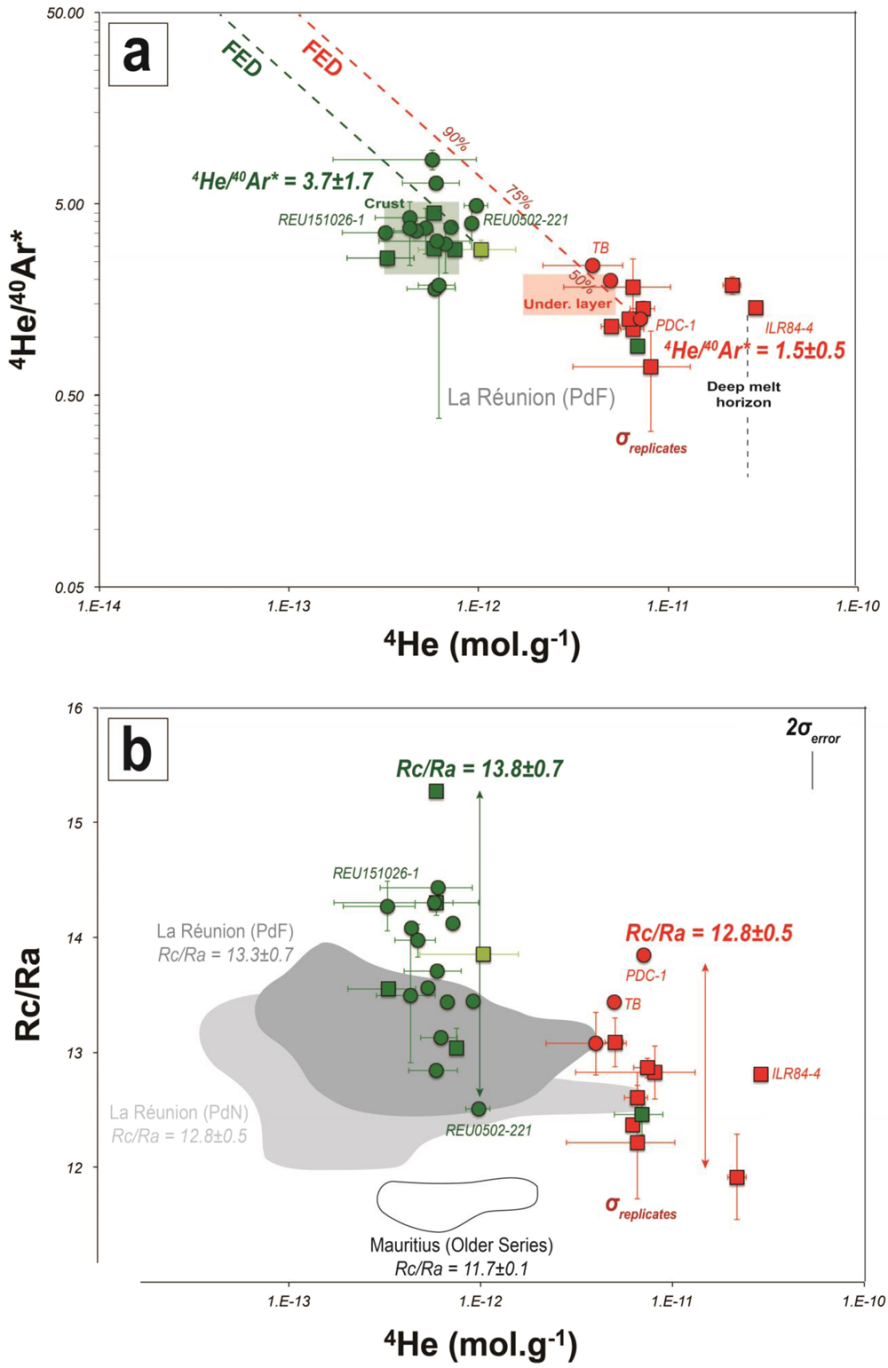
Gas contents and isotopes ratios obtained by crystal crushing from lavas and cumulates. (**a**) ^4^He concentration vs. ^4^He/^40^Ar*. FED for Fractional Equilibrium Degassing curves. See Methods for supplementary information on the modelled curves. (**b**) ^4^He vs. Rc/Ra (see Methods). Same legend than in Fig. 1 for the samples (circles and squares for analyses from crystals in lavas and cumulates, respectively). Dark and bright grey fields for published data at La Réunion island for lavas at PdF and at PdN, respectively. White field for published data at Mauritius island. Helium contents as a function of the part of the plumbing system (crust, underplating layer ‘under. layer’, deep melt horizon): squares for the full ranges of contents calculated (Fig. S1) from TGC values reported at PdF^10^. σ_error_ (bar in the corner) for the analytical uncertainty (on Rc/Ra). σ_replicates_ (individual bar for each dot) for the natural variability (standard deviation on the mean for each sample for which replicates were measured).

He concentrations (Fig. 2a) in FI in olivine range from 2.9 × 10^−11^ to 1.0 × 10^−12^ mol·g^−1^ for eccentric products (NWRZ) and decreases to 6.9 × 10^−12^ to 3.2 × 10^−13^ mol·g^−1^ for central cases (Enclos Fouqué, SERZ, NERZ). The correlation found here between the He and the total gas content (TGC) released by crystal crushing (Fig. S1) is consistent with the general view that central and eccentric products reflect entrapment pressures in the crust and in the upper mantle, respectively, and consequently record different extents of magmatic degassing^10^ (Table 1). In addition, ^4^He/^40^Ar*, which increases by fractional degassing at PdF during magma ascent^10^, displays higher values in central products (3.7 ± 1.7, on average) than in eccentric ones (1.5 ± 0.5, on average) (Table 1). These latter values are well above the ratio reported for primary degassed volatiles from the mantle beneath La Réunion island (^4^He/^40^Ar* = 0.3 ± 0.2)^10^. This increase further confirms the more evolved and degassed nature of central magmas as previously indicated by magma and mineral composition and fractional equilibrium degassing modeling^10^ (Figs. 2a and S1). Helium isotope ratio (^3^He/^4^He) corrected for atmospheric contamination (i.e., Rc/Ra; see Methods) varies from 11.9 to 15.3 Ra in FI from crystals of cumulate rocks and from 12.5 to 14.4 Ra in those of lavas (Fig. 2b). Our new and extended sampling reveals a greater variability in Rc/Ra than previously reported on the island^36,37^. More importantly, we found on average, higher values in central crustal products (Rc/Ra = 13.8 ± 0.7, on average) than for eccentric ones (Rc/Ra = 12.8 ± 0.5, on average), which record a deeper origin^10^. We highlight that the whole range of ^3^He/^4^He variability is not related to crystal size^38^ (all crushed crystals being >0.5 cm). No noticeable difference exists between the ^3^He/^4^He of the gas entrapped in FI from olivine or clinopyroxene crystals from wehrlites^39^ (Table S1).

Thermal (28–36 °C) springs (Irénée, Véronique, Manès) at Cilaos (Fig. 1) are located on PdN volcano, 30 km from the PdF summit^10,27,40,41^. Volcanic gases from these thermal springs are CO_2_-dominated^27^ (>90,000 μmol/mol; Table S2). Ratios of ^4^He/^40^Ar* vary from 1.3 to 1.4 (Irénée source only). These values are consistent with those from FI and are representative of the depth of the upper mantle, supporting the hypothesis of dominant exsolution and release from this depth^10^ (Table 1). In these gases, the two sampling campaigns performed between 2016 and 2017 revealed Rc/Ra variations from 13.1 to 13.6 at Irénée, between 13.4 and 14.0 at Véronique, and between 13.4 and 13.9 at Manès. Irrespective of the slight variation in Rc/Ra values between the three sources, it is important to highlight that the ^3^He/^4^He values measured in 2016–2017 are systematically higher (ΔRa = 0.7–1.5) than those reported in 1990 (i.e., Rc/Ra = 12.1 at Irénée, 12.7 at Véronique and 12.7 at Manès)^27^.

### Origin of helium isotope variability in fluid inclusions

**Figure 3:**
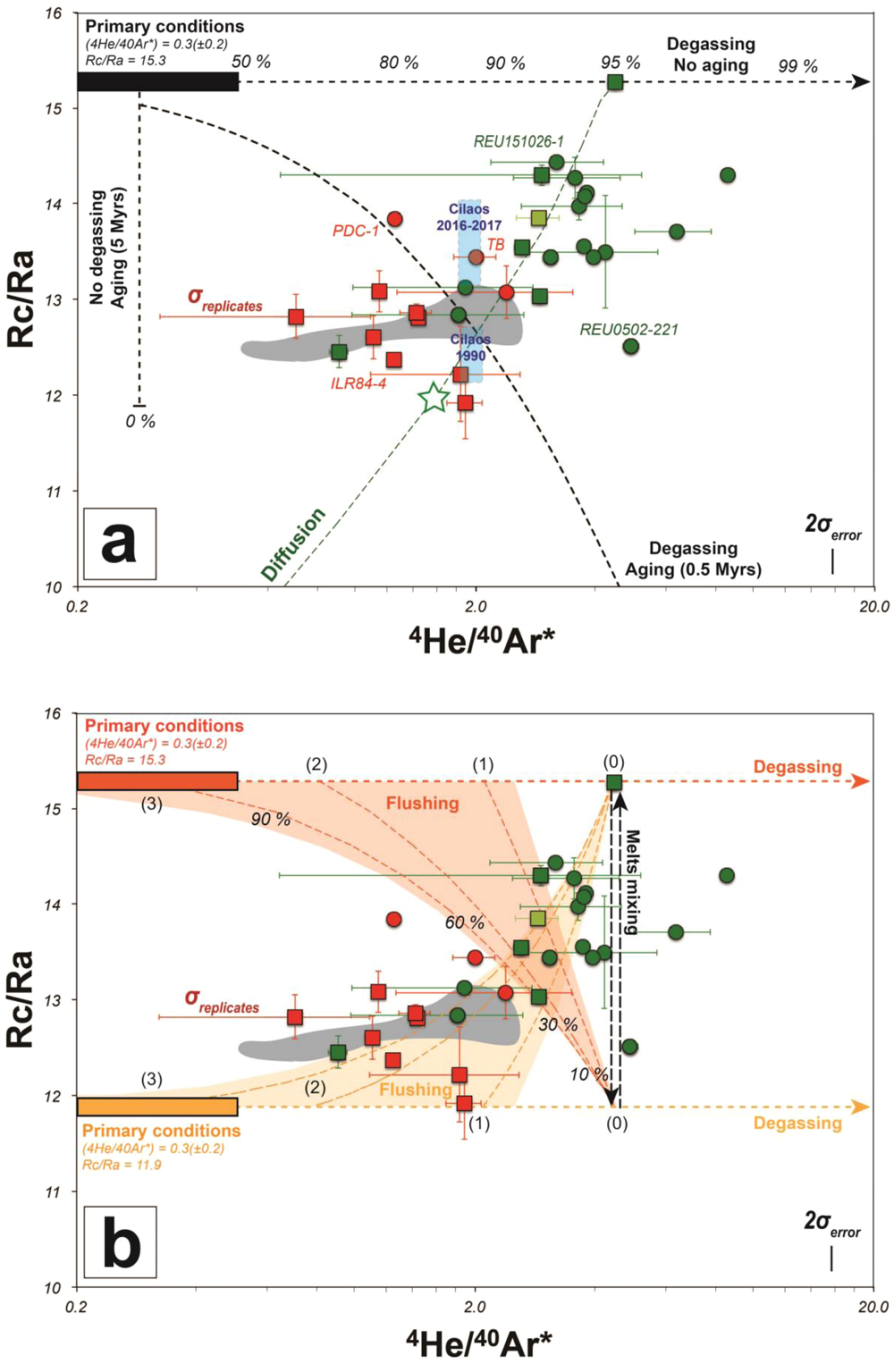
Noble gases isotopes ratios obtained by crustal crushing from lavas and cumulates. (**a**) ^4^He/^40^Ar* vs. Rc/Ra modelling the effect of diffusion (green dashed lines; the star highlights the effect of the diffusion on a crystal of 0.5 or 1 cm of diameter after 2 or 8 years, respectively) and the combined effect of magma aging and degassing (black dashed lines). The extent of degassing is reported (e.g., percentage). (**b**) ^4^He/^40^Ar* vs. Rc/Ra modelling the effect of degassing, gas flushing and melt mixing resulting from the melting/degassing of two distinct components of the mantle plume below La Réunion island. The part of the plumbing system where the volatile phase is exsolved (starting conditions) to generate gas flushing in the upper parts is characterised by its average ^4^He/^40^Ar* value^10^: (0) oceanic crust, (1) mantle-crust underplating layer, (2) deep melt horizon and (3) mantle source. The extent of gas flushing is reported (e.g., percentages). See Methods for supplementary information on the modelled curves. Same legend than for Fig. 2. Dark grey field for previous data at PdF. The meaning of σ_error_ and σ_replicates_ is detailed in the Methods and the caption of Fig. 2.

**Figure 4:**
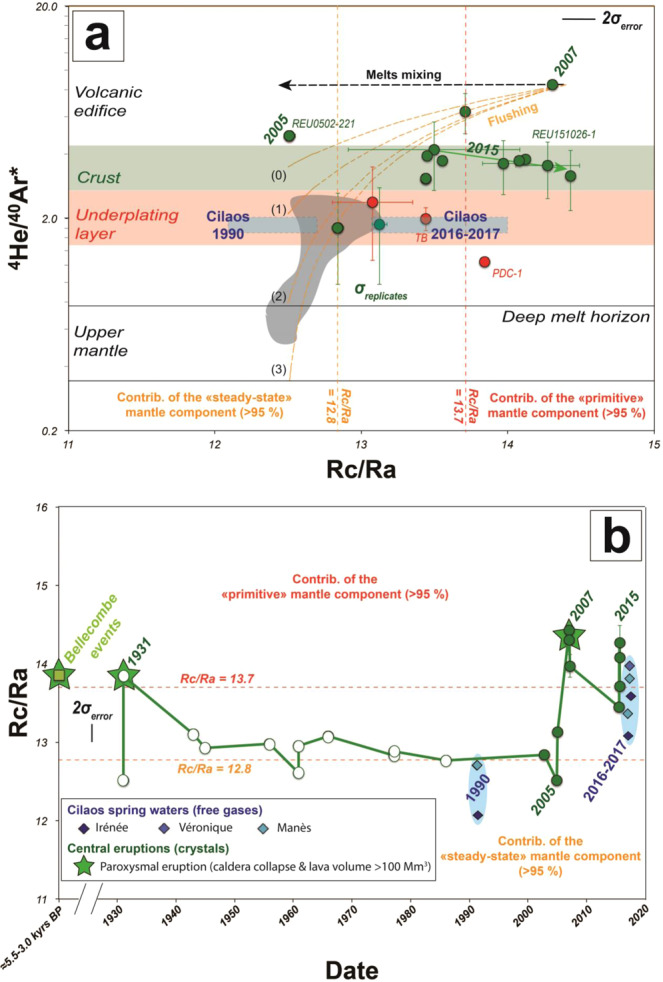
Noble gases isotopes ratios obtained by crystal crushing from lavas and compared to volcanic gases from thermal springs at Cilaos. (**a**) Rc/Ra vs. ^4^He/^40^Ar* modelling the effect of magmatic fluids flushing and melts mixing starting from the highest Rc/Ra vs. ^4^He/^40^Ar* values. Thresholds of the >95%-contribution of either the “primitive” mantle component or the “steady-state” mantle component to Rc/Ra value determined using the maximum-likelihood method^59^. See Methods for supplementary information on the modelled curves and the statistical method. Same legend than for Figs. 2 and 3. Blue field for the range of compositions from volcanic gases from thermal springs. Dark grey field for published data from lavas at PdF. The green arrow represents the chronology of the products from the August 2015 eruption. ^4^He/^40^Ar* variability as a function of the location of magma ponding in the plumbing system^10^. (**b**) Time variations of Rc/Ra from both FI in olivine crystals from PdF lavas and from volcanic gases. During the last century^30,57^, major eruptive events (caldera collapse and erupted volume >100 Mm^3^) occured only in 1931 and 2007. The meaning of σ_error_ and σ_replicates_ is detailed in the Methods and the caption of Fig. 2.

Previous studies, focused on noble gases from both eccentric and central products of PdF and PdN, have described a restricted range (ΔRa < 1) of ^3^He/^4^He values at La Réunion for at least the last million years^37^. Conversely, our new data highlight a greater variability (ΔRa = 3.4; Fig. 2b). The low variability documented in previous studies could mostly reflect the effect of the former sampling strategy focused on olivine rich “oceanite” basalts. Those basalts are characterized by phenocrysts mixed with antecrysts^28^. When “bulk” crystals are crushed for the extraction of FI, this mixing could lead to the homogenization of the ^3^He/^4^He signature of the different crystal populations, thus providing a narrower range of values. Conversely, we investigate products ranging from almost aphyric to olivine-rich basalts and cumulates. This sampling strategy may well explain why we observe a wider range of ^3^He/^4^He ratios than previous studies. This variability could be explained by different processes:

(1) Shallow crustal contamination. Although this is a possibility, the highest Rc/Ra values reported for central-crustal products with respect to eccentric-mantellic cases rule out shallow crustal contamination as the main process responsible for our ^3^He/^4^He variability. Similar conclusions were reported in recent studies based on Sr and Nd isotopes at PdF^42,43^ (Fig. 3a).

(2) Kinetic effects due to diffusive fractionation during melting of the mantle source region^9,44^. Considering the partition coefficients of He isotopes^39^, such a fractionation process would only have played limited role in our case because it should have led to both a decrease in ^3^He/^4^He and ^4^He/^40^Ar* in time, which is not observed. In addition, part of the ^3^He/^4^He variability occurs during a single eruption over a very short (days) time span (e.g., August 2015; Figs. 4 and 7; Table S1).

(3) Post-eruptive radiogenic ingrowth of ^4^He or cosmogenic ^3^He addition due to exposure to cosmic rays^45^. We can exclude such processes here due to the variability documented over the very short (eruptive) time spans. During the August 2015 eruption, the ^3^He/^4^He varied from 13.4 Ra (single measurement) on October 7, 2015 to 14.3 ± 0.2 Ra (on average) on October 26, 2015 (green arrow on Fig. 2b). Furthermore, the analytical method used in this work to release fluid inclusions (single-step crushing) minimizes the effect of post-eruptive ingrowth.

(4) Diffusive loss of noble gases out of crystals. This process may fit part of our dataset leading to lower Rc/Ra and ^4^He/^40^Ar* values^9,46^ after a few years (less than 8 years for crystals smaller than 1 cm; Fig. 3a). However, it cannot explain the synchronous variability found in our volcanic gas measurements and fluid inclusions (Table S2).

(5) Magmatic degassing coupled to aging. This process could decrease the ^3^He/^4^He values^6,12^. However, the lowest Rc/Ra values documented for eccentric mantle-derived products, i.e., for the most undegassed products with respect to central crustal cases^10^ are not consistent with the effect of magma degassing coupled to aging. We calculate that melts having degassed about 90% of their primary volatile content can decrease Rc/Ra from 15.3 to 11.9 only after 0.5 Myr of residence at the depth of the underplating layer^10^ (Fig. 3a). This time span is unrealistic considering that magma residence time at PdF is on timescale of thousands of years at maximum^37,47^. Conversely, residual material potentially ponding in the mantle melting region^48^ for about 5 Myr, i.e., in the range of ages proposed for the early construction of La Réunion island^49^, may decrease the Rc/Ra signature (Fig. 3a). In this case we would expect a progressive decrease of Rc/Ra values from the Older Series at Mauritius, through PdN to the youngest PdF products. However, this behaviour is not observed^50^.

Thus, we propose that the ^3^He/^4^He variability documented at PdF is related to the contribution of various extents of mixing of magmatic fluids coming from either a “high Rc/Ra” component that is identifyed in a few samples (e.g., “primitive” component in the following parts) or a “low Rc/Ra” component predominant in most samples from literature and our dataset (e.g., “steady-state” component in the following parts) (Fig. 3b). Similar cases of mixing between magmatic fluids coming from distinct components have been also reported at other hotspot volcanoes^1,45,51,52^. Our ^4^He/^40^Ar* data provide a new insight into the nature of this mixing of magmatic fluids. We document a greater variability in ^4^He/^40^Ar* for central crustal products with respect to eccentric cases and to previous studies performed at La Réunion^36,37,50,53^. This is irreconcilable with the effect of fractional equilibrium degassing only^10^ (Fig. 2a). Melt mixing and degassing10 should be expected to maintain constant ^4^He/^40^Ar* at constant pressure (Fig. 3b), unless primary melts originate from mantle portions that suffered different percentages of melt extraction. However, our results show that flushing by a gas phase exsolved at mantellic depths (e.g. (2) or (3) on Figs. 3b and 4a) is required to explain both the Rc/Ra and ^4^He/^40^Ar* variability observed in our dataset. At PdF, gas flushing is sustained by the presence of extensive degassing at the mantle level beneath La Réunion island^10^. Furthermore, the solubility of volatile elements in melts, together with the presence of multiple magma ponding zones and mixing steps at PdF^33^, can produce a migration of the gas phase across the plumbing system. This can occur faster than the rate of melt ascent, making the gas more susceptible to generating secondary FI^10,33,54^. This idea is supported by the ^3^He/^4^He, which is similar in the gas phase released by from both thermal springs and secondary FI in crystals from lavas emitted in the same period (e.g., 2015–2017; Table 1; Fig. 4a).

### “Paroxysmal” eruptions linked to a predominant contribution of magmatic fluids from a “primitive” mantle component

In historical times (post-1860 CE), discrete and short-lived effusive eruptions associated with low lava fountains, which quickly evolve into strombolian activity, represent the main eruptive dynamics at PdF^30,55^. During the period 1998–2010, i.e., one of the most recent and best documented eruptive periods of PdF, this “classical” eruptive style produced eruptions with average erupted volumes and durations of 6.6 Mm^3^ and 25 days, respectively^56^. This activity contrasts with rarer “paroxysmal” eruptions linked to caldera collapses and to the emission of unusually large volumes of lava^30,35,55,57^. Since 1930, two major “paroxysmal” eruptions have been observed^30,57^: in 1931 (130 Mm^3^)^57^and in 2007 (240 Mm^3^)^57^, both were associated with summit caldera collapses. Following the last “paroxysmal” eruption in 2007, a new phase of magmatic recharge of the central plumbing system began in 2014–2015^58^.

Since 2016, frequent sampling of the Cilaos thermal springs at PdN has revealed that the ^3^He/^4^He values (Rc/Ra = 13.5 ± 0.3, on average) in gases have increased with respect to those measured in 1990 at the same sites^27^ (Rc/Ra = 12.5 ± 0.3, on average; Fig. 4a). Importantly, our new sampling of eruptive products shows a similar increase in FI of products from syn- and post-April 2007 lavas (Rc/Ra = 14.0 ± 0.4, on average; Fig. 4b; Table 1) with respect to those from the 1986–2005 period (Rc/Ra = 12.8 ± 0.3, on average). The similarity of ^3^He/^4^He values in the gases related either to fluid inclusions or to thermal springs over time clearly indicates that ascent fluids are trapped in minerals in a short-time span that allows FI to preserve the same signature measured in volcanic gases. This result opens major prospects for future monitoring strategies. At PdF, the temporal evolution of Rc/Ra in gas released by crystal crushing from lavas emitted since 1931 shows two main events marked by Rc/Ra values greater than 13.7 (threshold value; see Methods): in 1931 and in April 2007 (and post-2007 products) (Fig. 4b). Together with products related to the Bellecombe’s explosive events (5.5–3.0 kyr BP; Rc/Ra = 14.3 ± 0.6, on average), these high ^3^He/^4^He values are linked to paroxysmal eruptions^30,35,57^ triggering caldera collapse and the emission of unusually high lava volumes (>100 Mm^3^). The 2007 paroxysmal eruption was triggered by a major input of deep magma that provoked the extrusion of a large amount of magma from the crustal plumbing system of PdF^58^. We thus infer that a predominant contribution (>95%) of the “primitive” mantle component leads to the production and ascent of large amount of melts and magmatic fluids with Rc/Ra >13.7 responsible of unusual paroxysmal eruptions. Conversely, the more frequent, small volume and short-lived eruptive activity at PdF would be related to a predominant contribution (>95%) of magmatic fluids from the “steady-state” component (Fig. 4b). This is the case of the February 2005 eruption (Rc/Ra from 12.5 to 13.1), i.e. the last eruption before the 2007 caldera collapse eruption from which we have get measurements of He isotopes.

### Changes in syn-eruptive magma dynamics deciphered using helium isotopes

**Figure 5:**
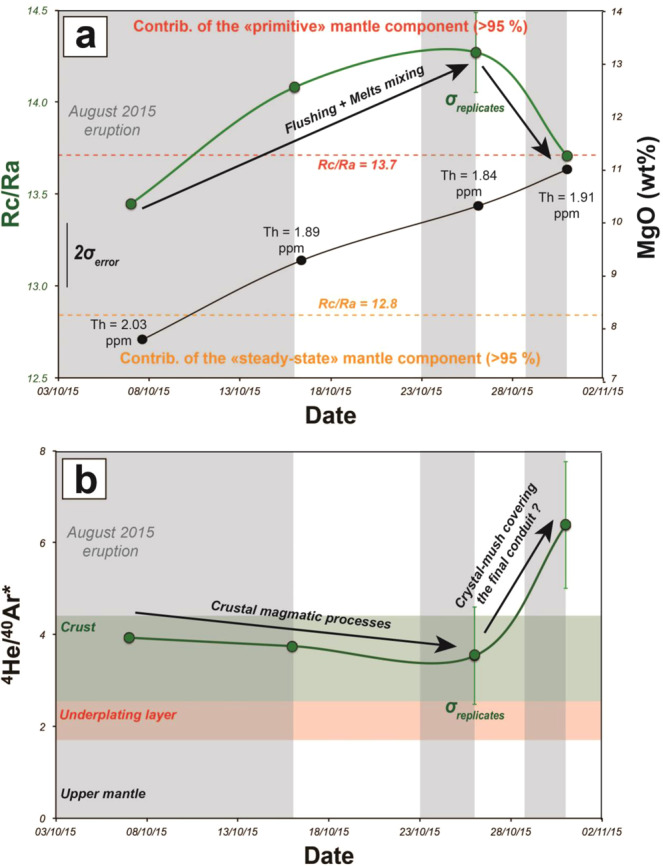
Time evolution of noble gases isotopes ratios in gas-released by olivine crystals crushing from lavas during the August 2015 eruption. (**a**) Evolution of ^3^He/^4^He compared to hosting lava chemistry^58^. (**b**) Evolution of ^4^He/^40^Ar*. ^4^He/^40^Ar* variability as a function of the location of magma ponding in the plumbing system^10^. Same legend than for Fig. 7. The meaning of σ_error_ and σ_replicates_ is detailed in the Methods and the caption of Fig. 2.

^3^He/^4^He may also be used to track rapid changes in magma dynamics during a single eruption, as occurred in August 24 – October 31, 2015 (Fig. 5). This eruption, one of the longest (2 months) since 1930 (and involving 45 ± 15 Mm^3^), evolved through three main phases each related to a distinct magma composition^58,59^. Frequent sampling permitted the identification of a progressive change in magma composition over time towards a more mafic composition (see MgO content on Fig. 5a) and a progressive enrichment of lavas in olivine phenocrysts which began in early October 2015. In addition, three distinct behaviours in He isotopes data were observed during the eruption:

Lava sampled in October 7 had an intermediate ^3^He/^4^He signature (Rc/Ra = 13.4; single measurement). Two interpretations may be considered. In the former case the Rc/Ra signature may be that of a high ^3^He/^4^He melt (>13.7 Ra), like those emitted in April 2007, still ponding at shallow level (^4^He/^40^Ar* = 3.9) for many years and flushed by a gas phase related to steady-state degassing conditions (<12.8 Ra; Fig. 4a). This possibility is supported by petrological evidences that the 2008–2014 eruptions were fed by a ‘April 2007’-derived melt that differentiated and cooled in the shallow part of the plumbing system^60^. However, the arrival of a high ^3^He/^4^He melt (>13.7 Ra) that progressively mixed with a steady-state melt already ponding at shallow level (<12.8 Ra) is also an arguable hypothesis considering the reverse zoning of a few host olivine phenocrysts^41^. In both cases, this “mixing” process between magmatic fluids having distinct isotopic signature can be favoured by the presence of vertically extended magma storage zone, where magma ponding favours melt homogenisation and fluid re-equilibration during ascent^10^ and fits most of our data (Fig. 4a). Such hybrid melts might be drained during the first phases of the eruption^58^.

Since mid-October 2015, a progressive increase in Rc/Ra at quite constant ^4^He/^40^Ar* value was observed (October 16 and 23, 2015; Fig. 5). We interpret this behaviour as the effect of syn-eruptive refilling of the crustal part of the plumbing system resulting in the emission of more primitive melts that triggered two successive short-lived eruptive phases on October 22–24 and October 29–31 (Fig. 4a). The increase in soil CO_2_ flux, plume SO_2_ emissions and the emission of more mafic melts (MgO-rich) hosting zoned olivine crystals from mid-October 2015 support our assumption^58,59,61^.

Lower ^3^He/^4^He values (Rc/Ra = 13.7; single measurement) but also highest ^4^He/^40^Ar* (= 6.4; single measurement) were obtained for crystals from lava erupted a few minutes before the end of the eruption. These values are consistent with the presence of cumulative olivine crystals entrained upon flushing of the upper part of the magma conduit^10,28,58^, i.e., having higher ^4^He/^40^Ar*. We propose that such flushing of crystals may be favoured by an increase in the final magma output rate linked to closure of the dyke. This assumption is supported by a lava overflow that also preceeded the end of the eruption by a few minutes, and emptying of the lava lake inside the main eruptive vent (OVPF report). The lowest Rc/Ra values for this sample are associated with an increase of MgO contents and olivine crystals in the host lavas, as also documented for the mid-October products, but with an increase in Th content (Fig. 5a). This distinct behaviour of magma chemistry in this final sample of the August-October 2015 eruption mirrors the distinct behaviour observed for He isotope.

This eruption demonstrates that variations of ^3^He/^4^He may be documented on timescale of days and are closely linked to the dynamics of syn-eruptive magma transfer.

### Constraints on the “primitive” and “steady-state” mantle components

Even if the ^3^He/^4^He variability that we document here may be related to various contributions of mantellic fluids from either a “primitive” component (data set for the “paroxysmal” eruptions) or a “steady-state” one (data set for the “classical” eruptions), the exact source of these two components is still unclear. This problem is even more intringuing considering that, if magmas at La Réunion are marked by the Dupal anomaly like most of the Ocean Island Basalts (OIBs) and Mid Ocean Ridge Basalts (MORBs) of the Indian Ocean^62^, the mantle source is considered extremely homogeneous with respect to other ocean basaltic islands^28,37,50,63,64^. Only minor variations of Sr-Nd-Hf isotopes and incompatible trace elements ratios have been reported and mostly attributed to a primary feature of La Réunion mantle plume^42,43,64–67^. We argue here that the ^3^He/^4^He scale of variability (ΔRa greater than 3 units Ra) is also a primary feature of the mantle source beneath La Réunion island. Such an idea was previously developed for Hawaii, where ^3^He/^4^He fluctuations up to 8 Ra on a timescale of a few hundred years were attributed to the melting of primary heterogeneities of the mantle plume^14^.

**Figure 6:**
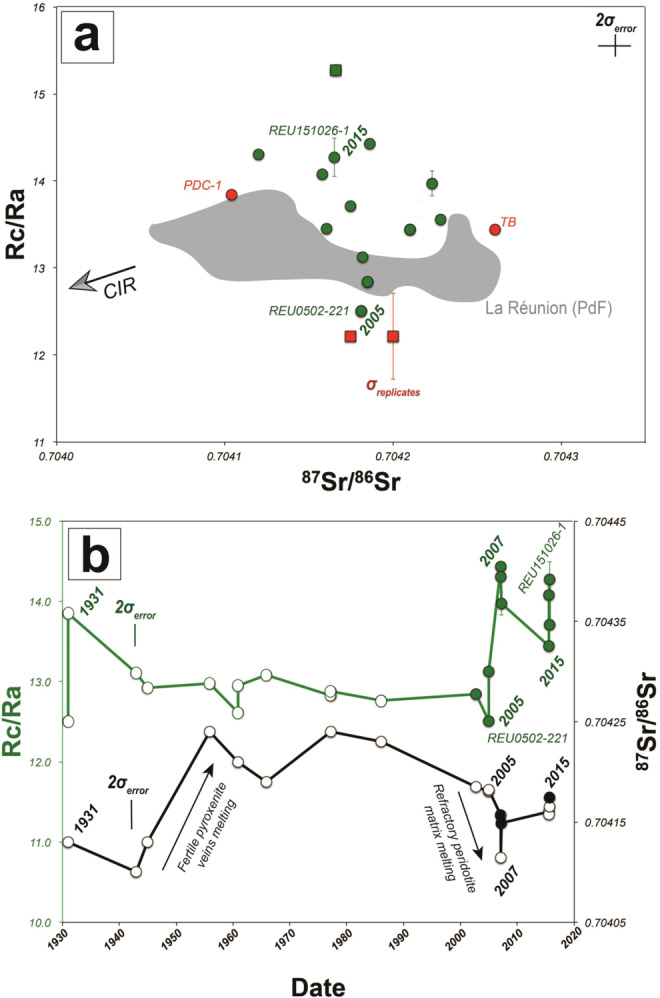
Comparison between helium isotopes (^3^He/^4^He) and strontium isotopes (^87^Sr/^86^Sr) at PdF. (**a**) ^87^Sr/^86^Sr vs. ^3^He/^4^He. Dark grey field for published data from lavas at PdF^37^. CIR for the Central Indian Ridge composition^72^. Same legend than in Fig. 1 for our samples. (**b**) Time evolution of ^3^He/^4^He and ^87^Sr/^86^Sr since 1931 from lavas. ^3^He/^4^He datasets before 2002 from previous studies^36,37^. ^3^He/^4^He after 2002 acquired in this study. ^87^Sr/^86^Sr dataset completed^37^ in this study for products emitted after 2002. New data are highlighted by filled circles. The meaning of σ_error_ and σ_replicates_ is detailed in the Methods and the caption of Fig. 2.

In the Indian Ocean, lower ^3^He/^4^He values were measured at Rodrigues and at Mauritius (Younger Series) and attributed to ridge-hot spot interaction, i.e., related to a contribution of magmatic fluids with a lower ^3^He/^4^He signature from the Central Indian Ridge (CIR) with respect to the mantle plume (Fig. 6a)^45,50^. At PdF, we do not find any correlation between ^3^He/^4^He and ^87^Sr/^86^Sr (Table 1; Fig. 6a) thus suggesting mixing between such distinct reservoirs^1,13,14,37,52,67,68^. Instead, decade-scale fluctuations of Sr isotopes have been documented for recent PdF lavas and attributed to the fertility of the source and induced melt channelization^43^ (Fig. 6b). In view of the absence of correlation between He and Sr isotopes, we propose that the ^3^He/^4^He decrease in some PdF products may be related to intrinsic features of the mantle plume beneath La Réunion island. This fits with other recent hypothesis showing that the composition of La Réunion mantle plume may derive from a mantle source that experienced an early-stage (Hadean) of differentiation generating distinct reservoirs or blobs within the mantle plume^37,42,64^. Similar episodic entrainment by thermal plumes of deep isolated mantle reservoirs of evolved versus primordial materials was previously reported in ocean island basalts^69^. In this context, the suspected global ^3^He/^4^He increase from Older Series at Mauritius (not contaminated by the Central Indian Ridge) (Rc/Ra = 11.7 ± 0.1, on average; Fig. 2b) to present-day products at PdF (Rc/Ra = 13.3 ± 0.7, on average; Fig. 2b) may reflect such progressive melting of the more primitive component of the mantle plume^50^.

On the other hand, the composition of some melts at PdF^33,65^ has suggested the presence of magmatic fluids enriched in U-Th, down to the upper mantle, which may be responsible for hydrothermal contamination in the PdF plumbing system^10,33,65^. The enrichment in U-Th in such magmatic fluids suggests a potential enrichment in ^4^He by radioactive decay in these fluids, which may lower the ^3^He/^4^He signature in some PdF products. For instance, we have reported a lower averaged Rc/Ra signature (Rc/Ra = 12.8 ± 0.5, on average) in eccentric products with respect to central crustal cases (Rc/Ra = 13.8 ± 0.7, on average), i.e., for products testifying to magma ponding at the depth of the mantle/crust underplating layer and even deeper (Fig. 2b). Such magma ponding, mixing and degassing are known to be important processes occurring in the upper mantle beneath La Réunion island^33,41^. Thus, in addition to some mantle plume heterogeneities, we cannot exclude the role of contamination by ^4^He-U-Th-rich magmatic fluids in the lithospheric mantle able to reduce the ^3^He/^4^He signature of melts ponding in the plumbing system^10,65^. Such decoupled ascent between ^4^He-U-Th-rich magmatic fluids and melts could be an additional argument supporting the apparent decoupling between He isotopes (gas in FI and volcanic gases) and Sr isotopes (melt and crystal phase) (Fig. 6).

## Conclusion

**Figure 7:**
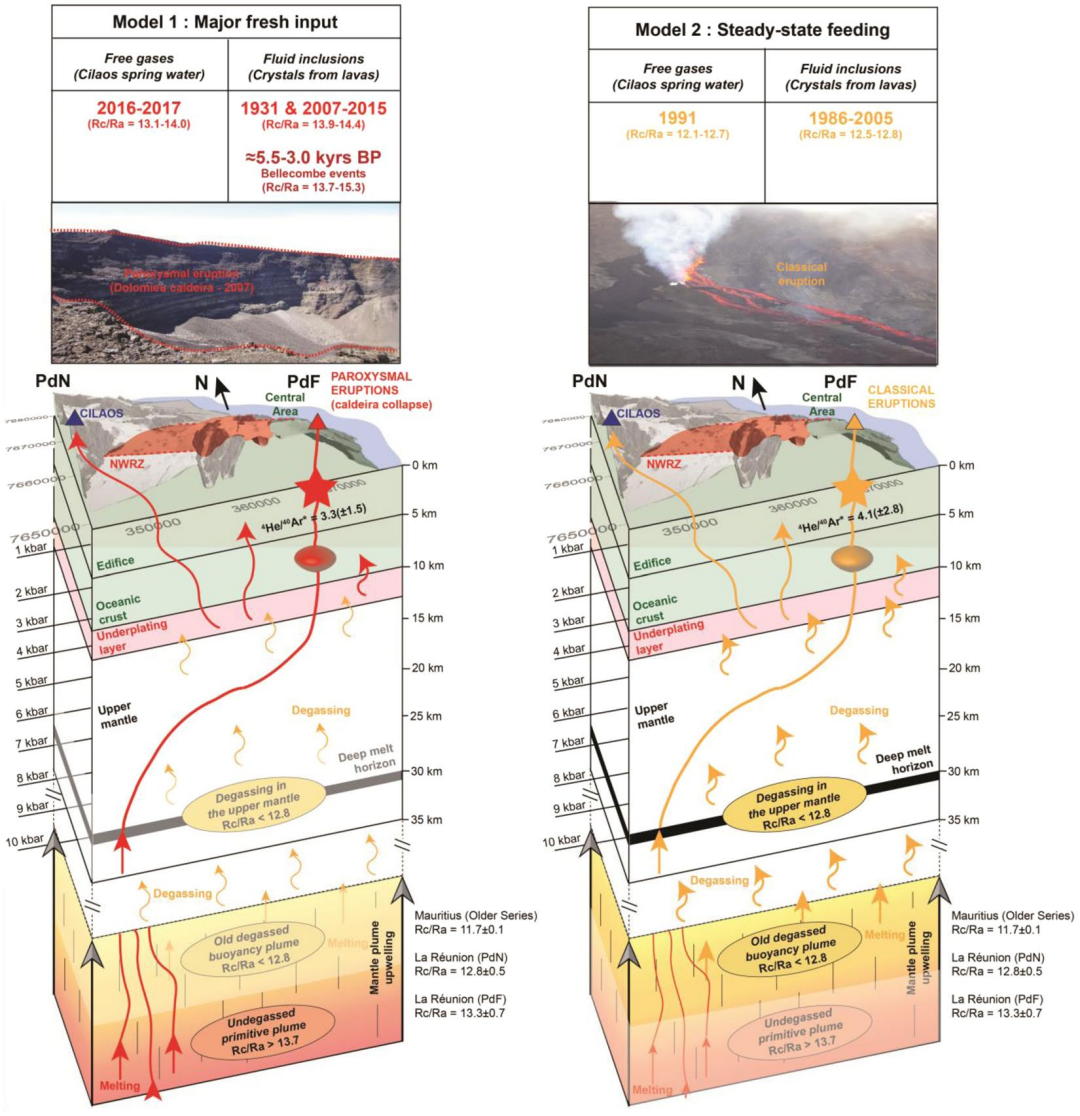
Conceptual model of the magmatic plumbing system of PdF^40,61^. Model 1 for major fresh melt input leading to « paroxysmal » eruptions as observed in 1931 and 2007 caldera collapse eruptions (Rc/Ra >13.5 in both FI and volcanic gases). There, magmatic fluids are mainly related to a primitive component of the mantle plume (Rc/Ra >13.7). Model 2 for steady-state melt feeding leading to « classical » eruptions as observed in 2005 (Rc/Ra <12.8 in both FI and volcanic gases). There, magmatic fluids are mainly related to an old degassed buoyancy component of the mantle plume (Rc/Ra <12.8) and/or to the contribution of ^4^He-rich magmatic fluids from the upper mantle. Pictures from the first author (CC BY 4.0).

Through the study of the ^3^He/^4^He signature of volcanic gases and fluid inclusions at La Réunion island, we infer that a predominant contribution (>95%) of a primitive component of the mantle plume leads to the production and ascent of melts and magmatic fluids (Rc/Ra >13.7) responsible for unusual “paroxysmal” eruptions, as observed for the most recent caldera collapse eruptions at Piton de la Fournaise (PdF) (Fig. 7). Conversely, the more frequent, small volume and short-lived eruptive activity at PdF would be related to (i) a predominant contribution (>95%) of magmatic fluids from a more degassed mantle component and/or (ii) greater residence times for melts that may be progressively contaminated by ^4^He-rich magmatic fluids coming from the lithosphere and producing steady-state magmatic conditions (Rc/Ra <12.8). These steady-state conditions are well recorded in “classical” eruptions such as that of 2005 (Fig. 7).

Our results suggest that the eruptive activity at hotspot volcanoes may respond to fast changes in mantle dynamics and lead to “paroxysmal” eruptions with serious implications in terms of hazard^70^. We emphasize that ^3^He/^4^He monitoring in volcanic gases may forecast the arrival of magmatic fluids triggering “paroxysmal” eruptions potentially related to caldera collapses, as observed at PdF (2007) and at Kilauea^25^ (2018). Considering the synchronous variations of ^3^He/^4^He values observed in volcanic gases and in gas from FI, our study opens exciting perspectives for assessing magmatic unrest, as well as syn-eruptive changes in magma dynamics, beneath more hazardous volcanoes globally. Our study supports the idea that coupling petrology (fluid inclusions barometry and petrography) with gas geochemistry (He-Ar isotopes) is fundamental to understand the variability of helium isotopes and their use for volcano monitoring.

## Methods

### Sr isotopes measurements

8 analyses of Sr isotopes ratios (^87^Sr/^86^Sr) have been performed at INGV-OV (Istituto Nazionale di Geofisica e Vulcanologia – Osservatorio Vesuviano di Napoli, Italy). Isotopic compositions have been determined on ca. 0.1 grams of crystal powders, residues of the crushing for He isotopes analyses, after dissolution with ultrapure acids (HF-HNO_3_-HCl mixtures). Sr was separated from the mineral matrix through conventional ion-exchange procedures (standard separation scheme^71^ for details) and measured statically by thermal ionization mass-spectrometry (TIMS, Thermo Finnigan Triton TI). During collection of isotopic data, replicate analyses of NIST-SRM 987 (SrCO_3_) international reference standards were measured. Mean measured values for ^87^Sr/^86^Sr for the NIST-SRM 987 standard were 0.710219 ± 0.000018 (2σ, N = 101). Measured ^87^Sr/^86^Sr have been normalized for within-run isotopic fractionation to ^86^Sr/^88^Sr = 0.1194 and have been normalized to the recommended value of ^87^Sr/^86^Sr = 0.71025 for NIST-SRM 987 standard. On products sampled the same day during the August 2015 eruption (REU151031) we found a difference <0.000027 on ^87^Sr/^86^Sr signature (i.e. in the range of the 2σ uncertainty on measurements) between our analysis performed in olivine crystals and those previously performed from bulk rocks^43^. Additionally, we have reported (Table S1) 8 analyses of Sr isotopes ratios (^87^Sr/^86^Sr) from literature performed on the same products that have been analysed in this study for noble gases^41,43^.

### Noble gases measurements

Samples analysed in our study from the central area spans a recent eruptive period (2002 to 2015). Eccentric ones were mostly erupted after the Bellecombe explosive events generating the Enclos Fouqué caldera^35^ (≈5.5–3.0 kyr BP). Olivine crystals without impurities (as well as clinopyroxene crystals for two wehrlitic cumulates) were handpicked from 30 samples previously crushed and sieved in the fractions of 0.5 and 1 mm. Minerals were first cleaned ultrasonically in 6.5% HNO_3_, second in deionized water and third in suprapure acetone. After being carefully weighed, the samples were loaded into a stainless steel crusher that allows to holding up to six samples simultaneously for noble gas extraction. FI were released by in-vacuo single-step crushing of minerals after that about 200 bar-pressure was applied by a hydraulic press. This procedure strongly reduces or avoids that cosmogenic ^3^He and radiogenic ^4^He that could possibly have grown or been trapped in the crystal lattice are released together with FI^11,72–75^. However, since our samples are very recent, any cosmogenic effect can be thus excluded. We determined the Total Gas Content (TGC) during crushing and FI extraction by measuring the total gas pressure (CO_2_ + N_2_ + O_2_ + noble gases). Then, a “cold finger” present in the crusher was immersed in liquid N_2_ at −196 °C to remove CO_2_ (and H_2_O, if present). The residual pressure of N_2_ + O_2_ + noble gases was quantified and subtracted from the TGC. The gas mixture was then purified under getters in an ultra-high-vacuum (10^−9^–10^−10^ mbar) line in order to leave only noble gases. A cold finger with active charcoal immersed in liquid N_2_ removed Ar, while He and Ne were separated by using a cold head preventively cooled at 10 K and then moved at 40 and 80 K by a temperature controller connected to a heater around the cold head in order to release He and Ne, respectively. A similar procedure was adopted in previous studies^11,75^.

Helium (^3^He and ^4^He) and neon (^20^Ne) isotopes were measured separately by two different split-flight-tube mass spectrometers (Helix SFT-Thermo). Argon isotopes (^36^Ar, ^38^Ar, and ^40^Ar) were analysed by a multicollector mass spectrometer (GVI Argus)^10^. The analytical uncertainty in the measured concentrations of ^4^He, ^20^Ne and ^40^Ar was <0.1% (analytical uncertainty reported as σ). Typical blanks for ^4^He, ^20^Ne and ^40^Ar were <10^−16^, <10^−16^, and <10^−14^ mol, respectively, which implies that blank incides on the sample peaks at the mass spectrometers of the above atomic mass units for <0.03%, <6%, and <0.4%, respectively. The protocol for the preparation, single-step crushing and analysis of FI was the same as that applied for other studies at the INGV, Sezione di Palermo (Italy)^10,11^. We do not document relevant shift between noble gases measurements performed in this study and previous measurements performed in another laboratory on the same sample (i.e. ILR84–4)^36,37^, which supports the accuracy of our measurements. Literature data at La Réunion island (PdF and PdN) and from Mauritius island are compared to our results^36,37,50,76^. At Mauritius, only results from the Older Series are reported due to their direct link with the mantle plume with respect to Youngers Series being MORB-contaminated^45,50^. In total, 72 measurements have been carried out for noble gases and TGC. A variable aliquot ranging from 0.08 g to 1.36 g of crystals was analysed in replicate measurements for the elemental and isotope composition of helium, neon, and argon in the gas trapped in FI (between 1 and 9 replicates per sample; the 9 replicates for PDC-1 represent a total weight up to 9.26 g of crystals). Analytical results are reported in Table S1. The analytical uncertainty of single measurements is reported as “σ_error_” for Rc/Ra and ^40^Ar/^36^Ar in Table S1 and figures. The natural variability of replicates (e.g. reproducibility) is reported as “σ_replicates_” (standard deviation on the mean of the measurements obtained for each replicates) for either the elemental concentrations or for the isotopic ratios. These analyses were performed in the noble gas isotope laboratory of INGV, Sezione di Palermo (Italy).

Bubbling gaseous samples from Cilaos thermal springs were collected as follows: because thermal springs are captured by three different plexiglass cups, gas was directly pumped from the vent placed at the top of each cup into a 100 cm^3^ syringe and then injected into the sample containers (with volume about 20–30 cm^3^) by using three-way valve. Air was purged away from containers by means of several cycles of pumping and injection. Two-ways Pyrex bottles, with vacuum valves at both ends, were used as containers for measurements of major and reactive gases. Two-ways stainless steel tube, with swagelok valves at both ends, was instead employed to sample gas for noble gas analyses. In all samples, the concentrations of CO_2_, CH_4_, N_2_, O_2_, He, CO, and H_2_ were routinely measured at INGV, Sezione di Palermo (Italy) by a Perkin Elmer Clarus 500 gas chromatograph equipped with a 3.5-m Carboxen 1000 column and double detector (hot-wire detector and flame ionization detector), with analytical errors of <3%^18,26,77^. Gas aliquots for noble gas analyses were introduced into three distinct stainless-steel ultra-high-vacuum lines for standard purification procedures to remove major and reactive species. In detail, each of the three preparation lines is dedicated to a single noble gas species, in our case He, Ne, and Ar, and is equipped with a pipette system connected to a manometer to introduce a known number of moles of gas sample. The purification procedure for helium, neon, and argon is in principle the same adopted for fluid inclusions above described and reported in previous studies for free gases^11,21,77^. The abundances and isotope compositions of He were determined by a split flight tube mass spectrometer (Helix SFT-GVI). Neon abundance and isotope composition (^20^Ne) was determined by a Helix MC Plus Thermo. The abundances and isotope compositions of Ar were measured in a multicollector mass spectrometer (Helix MC-GVI). The analytical errors of the He, Ne, and Ar-isotope analyses were less than 0.4%, 0.06% and 0.1%, respectively. Internal standard was purified from air, whose ^3^He/^4^He reproducibility over 1 year of daily analysis was <3.5%, ^20^Ne reproducibility over 1 year of daily analysis was <4%, ^40^Ar/^36^Ar reproducibility over 1 year of daily analysis was <3.5%. Typical blanks for He, Ne, and Ar were <10^−16^, <10^−16^ mol, and <10^−14^ mol, respectively, being at least two orders of magnitude lower than sample signals at the mass spectrometers. Detailed measurements were performed in October 2016 and April 2017 for the 3 sources of Cilaos thermal springs (Irénée, Véronique, Manès). Historical (1990) data from the same sources are compared to our results^27^.

### Noble gases corrections from the atmospheric component

A correction from atmospheric contamination is necessary in order to obtain the so-called magmatic-derived ^40^Ar*. This has been calculated, accordingly with the following formula that assumes that all the measured ^36^Ar is of atmospheric origin:

$$40A{r}^{\ast }=40A{r}_{m}-{\left(\frac{40Ar}{36Ar}\right)}_{air}\times 36A{r}_{m}$$

where ^40^Ar* represents the corrected ^40^Ar (^40^Ar/^36^Ar = 295.5), and the “m” subscript indicates “measured”.

The ^3^He/^4^He ratio is expressed as R/Ra (being Ra the He isotope ratio of air and equal to 1.39 × 10^−6^) and was corrected from atmospheric contamination following the equation^78^:

$$Rc/Ra=\frac{(R\times N-Ra\times Na)}{(N-Na)}$$

where Rc/Ra, R and Ra are ^3^He/^4^He of the corrected, observed and atmospheric components, while N and Na are ^4^He/^20^Ne measured and atmospheric components (Na = 0.318), respectively. The analytical uncertainty (σ_error_ reported in Table S1) does not exceed 0.2 Ra on average.

### Noble gases modelling

Various fractionation processes were used to model the noble gases variability described in our study. We here detail the equations and parameters used to perform this modelling.

1. Fractional equilibrium degassing
  Fractional equilibrium degassing (FED) was modelled from the equation^79^:
  $${\left(\frac{{}_{v}{}^{4}He}{{}_{v}{}^{40}A{r}^{\ast }}\right)}_{FED}={\left(\frac{{}_{l}{}^{4}He}{{}_{l}{}^{40}A{r}^{\ast }}\right)}_{ini}\times \alpha \times {F}^{\alpha -1}$$
  where ${({}_{v}{}^{4}He/{}_{v}{}^{40}A{r}^{\ast })}_{FED}$ is the ratio in the vapor phase following a FED trajectory, ${({}_{l}{}^{4}He/{}_{l}{}^{40}A{r}^{\ast })}_{ini}$ is the initial ratio in the melt, *F* is the fraction of residual gas (between 1 and 0) and *α* is the Ar/He relative solubility. In Fig. 2, ${({}_{l}{}^{4}He/{}_{l}{}^{40}A{r}^{\ast })}_{ini}$ is equal to 12.7 and 27 for eccentric (e.g. PDC-1; red) and central (e.g. ol; green) products and assuming a Ar/He solubility of 0.10 and 0.11, respectively^10^. In Fig. S1, ${({}_{l}{}^{4}He/{}_{l}{}^{40}A{r}^{\ast })}_{ini}$ range from 1 to 5 for FED modeling in primary conditions^10^ and assuming a Ar/He solubility of 0.10. The same equation is also used in Fig. S1 substituting ${({}_{l}{}^{4}He/C{O}_{2})}_{FED}$ to ${({}_{l}{}^{4}He/C{O}_{2})}_{FED}$ and assuming that ${({}_{l}{}^{4}He/C{O}_{2})}_{ini}$varies from 1.2 × 10^−5^ to 2.2 × 10^−5^ and the He/CO_2_ solubility^10^ equal to 1.7.
2. Diffusion
  To model noble gases diffusion across crystals we have made the assumption that crystals can be considered like perfect spheres using the equation^80^:
  $$\frac{C(t,r)-{C}_{1}}{{C}_{0}-{C}_{1}}=1+\frac{2\times a}{\pi \times r}\times \mathop{\sum }\limits_{n=1}^{\infty }\frac{{(-1)}^{n}}{n}\times \,\sin (\frac{n\times \pi \times r}{a})\times \exp (\frac{-D\times {n}^{2}\times {\pi }^{2}\times t}{{a}^{2}})$$
  where *C*_1_ is the initial concentration of the sphere (here the studied gas content in the crystal, i.e. ^3^He, ^4^He or ^40^Ar*), *C*_*o*_ is the surface concentration (here considered null for modelling noble gases diffusion out of the crystal)^9^, *a* is the half-diameter of the sphere (here varying between 0.25 and 0.5 cm in accordance with the size of the crystals analysed in this study), *t* the time (in s) and *r* the distance from the center of the crystal (here r = $a/{2}^{\frac{1}{3}}$ in order to consider *C(t,r)* as the average concentration of the sphere. The equation was resolved using a homemade script in Python 2.7 language using the SciPy, NumPy and math libraries. Diffusion coefficients *D* across olivine crystals were set at 1.61 × 10^−10^, 1.85 × 10^−10^ and 5.09 × 10^−11^ cm^2^/s for ^4^He, ^3^He and ^40^Ar, respectively^9,46^. Initial conditions were fixed at Rc/Ra = 15.3 (maximum value in our dataset; e.g., CUM2) and ^4^He/^40^Ar* varying from 4.5 (e.g., CUM2) to 10 inside the crystal and at 0 outside (i.e. negligible in the surrounding melt with respect to the crystal like proposed in previous modelings^7^).
3. Aging
  The effect of radiogenic ^4^He production from the decay of U and Th over time (^4^He*) can be estimated according to the equation^81^:
  $$4H{e}^{\ast }=2.80\times {10}^{-8}\times [U]\times (4.35+\frac{Th}{U})\times t(c{m}^{3}STP/g)$$
  where t is the time in Ma, [U] is the uranium concentration (0.5 ppm on average in PdF melts)^41^ and Th/U is the thorium-uranium atomic ratio (3.9 for La Réunion)^45,47,82^. We estimate the resulting ^4^He* production at 5.18 × 10^−18^  ol/g per year. Initial conditions were set at ^4^He/^40^Ar* = 0.3 (primary conditions from the first exsolved phase)^10^ and Rc/Ra = 15.3 (maximum value in our dataset; e.g., CUM2). We have modelled (i) melt degassing (F varying from 0 to 1 to represent the extent of FED) without aging, (ii) melt degassing with aging (0.5 Myrs i.e. similar to the age of the beginning of the PdF subaerial volcanic activity)^49^ and, (iii) melt without degassing but with the effect of aging (5 Myrs i.e. related to the end of the Older Series at Mauritius island and at the beginning of the volcanic activity at La Réunion island)^49,50^.
4. Mixing and statistical analysis

On Fig. 3, gas flushing was modelled as an arrival of a deeper magmatic gaseous phase mixing with the volatile phase already exsolved at the depth of magma ponding. We here modelled through isotopic and mass balances equations the effect of gas flushing of a gas phase similar to that of CUM2 (^4^He/^40^Ar* = 4.5 i.e. ponding at crustal level), which have the highest ^3^He/^4^He ratio of our dataset (e.g., 15.3 Ra), with a volatile phase arriving from the deep melt horizon or the underplating layer with the lowest ^3^He/^4^He signature found in our dataset (e.g., 11.9 Ra) and ^4^He/^40^Ar* = 0.3, 0.8 and 2.1^10^. The orange field shows the whole field of theorical evolution of the gaseous phase considering the uncertainty relative to ^4^He/^40^Ar* signatures^10^. The inverse process (e.g. gas flushing through the arrival of a volatile phase with the highest Rc/Ra signature in a gas phase marked by the lowest Rc/Ra signature) is also modelled with the red field and curves. On the contrary, melt mixing is expected to product a ^3^He/^4^He variability in the exsolved phase at constant ^4^He/^40^Ar*.

On Fig. 4, we have modelled the effect of gas flushing (with a ^3^He/^4^He signature of 12.5 Ra, i.e. similar to the minimum signature in our dataset for lava samples: REU0402-221 from the February 2005 eruption) on a volatile phase marked by the highest Rc/Ra signature (e.g., 14.4 Ra in REU0704-52 from the April 2007 eruption) and the highest ^4^He/^40^Ar* value (e.g. 8.5 in REU0704-51 from the April 2007 eruption) in our dataset for lava samples.

Thresholds of the contribution (>95%) of either the “primitive” (^3^He/^4^He > 13.7 Ra) or “steady-state” (^3^He/^4^He < 12.8 Ra) mantle component were determined from the 47 analysis obtained from PdF lava samples (our study and literature; Table S1) with the maximum-likelihood method using a Gaussian Mixture Model (GMM) implementing an expectation-maximization (EM) algorithm developed in Python 2.7 language with the NumPy and sklearn libraries^59,83^.

## Supplementary information

Supplementary Information1.

Supplementary Information2.

Supplementary Information3.
