# Supplementary material for: Paroxysmal eruptions tracked by variations of helium isotopes: inferences from Piton de la Fournaise (La Réunion island)
Source: Sci Rep. 2020 Jun 17;10:9809. doi: 10.1038/s41598-020-66260-x (PMC7300000; doi:10.1038/s41598-020-66260-x)
Supplement: Supplementary file 1 — Supplementary Information1. [file 41598_2020_66260_MOESM1_ESM.pdf]

Paroxysmal eruptions tracked by variations of helium isotopes: inferences from Piton de la Fournaise (La Réunion island)

G. BOUDOIRE[1,2]*, A.L. RIZZO[2,3], I. ARIENZO[4], A. DI MURO[5,6]

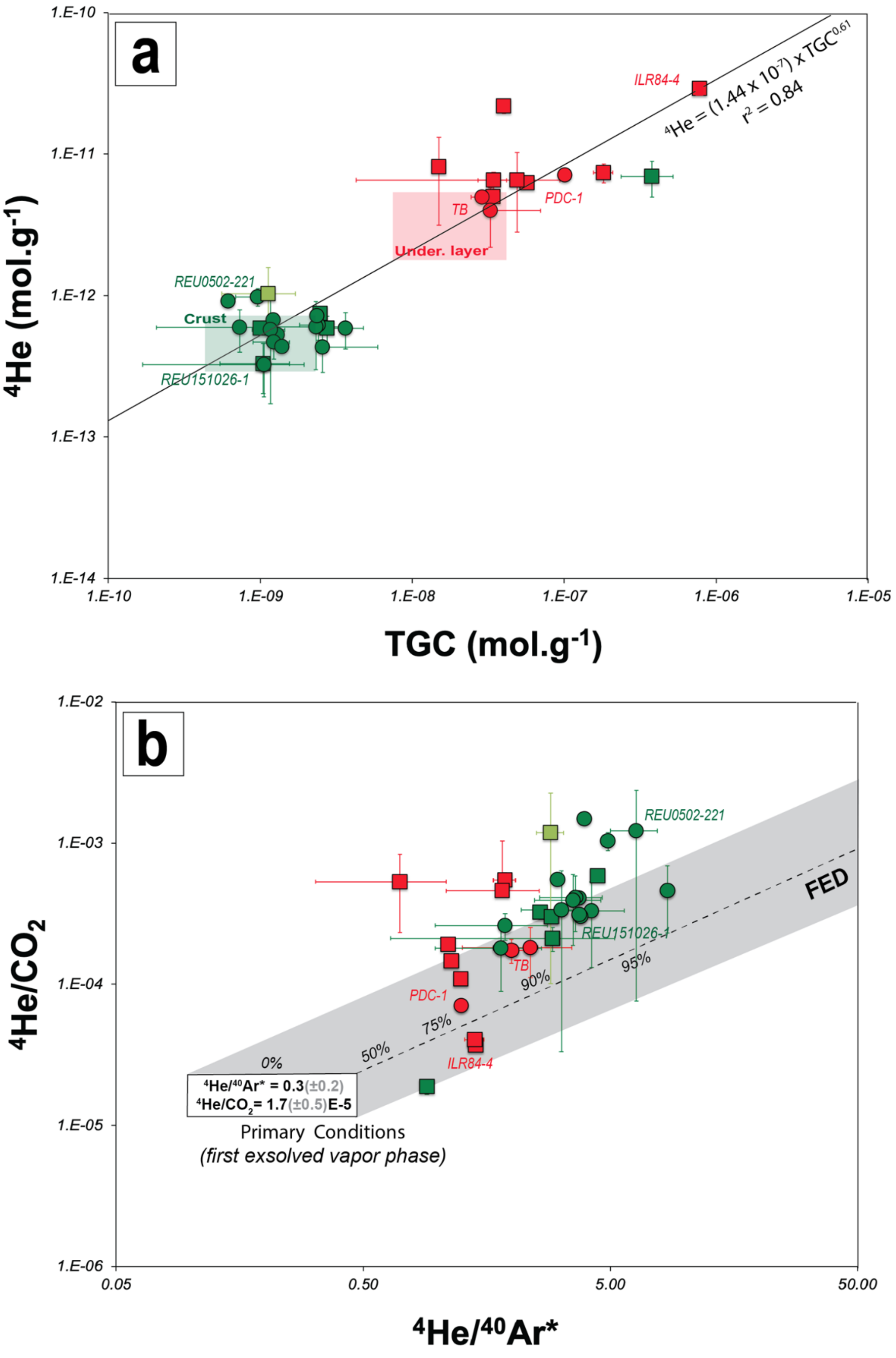

Fig. S1. Gas contents and isotopes ratios obtained by crystal crushing from lavas and cumulates. (a) TGC (Total Gas Content) vs. ^4He. TGC variability as a function of the location of magma ponding in the plumbing system (crust, underplating layer, deep melt horizon)[10]: squares for the full ranges of contents. Correlation between TGC and ^4He contents calculated from the whole dataset. (b) $^4He/^{40}Ar^*$ vs. $^4He/CO_2$ with FED (Fractional Equilibrium Degassing) modeling[10]. Grey field for the range of uncertainty related to the primary conditions. Same legend than in Fig. 1 for the samples.
